# Reverse translated and gold standard continuous performance tests predict global cognitive performance in schizophrenia

**DOI:** 10.1038/s41398-018-0127-5

**Published:** 2018-04-12

**Authors:** Andrew W. Bismark, Michael L. Thomas, Melissa Tarasenko, Alexandra L. Shiluk, Sonia Y. Rackelmann, Jared W. Young, Gregory A. Light

**Affiliations:** 1 VISN-22 Mental Illness Research Education and Clinical Center (MIRECC), VA San Diego Healthcare System, San Diego, USA; 2 Department of Psychiatry, University of California, San Diego, USA

## Abstract

Attentional dysfunction contributes to functional impairments in schizophrenia (SZ). Sustained attention is typically assessed via continuous performance tasks (CPTs), though many CPTs have limited cross-species translational validity and place demands on additional cognitive domains. A reverse-translated 5-Choice Continuous Performance Task (5C-CPT) for human testing—originally developed for use in rodents—was designed to minimize demands on perceptual, visual learning, processing speed, or working memory functions. To-date, no studies have validated the 5C-CPT against gold standard attentional measures nor evaluated how 5C-CPT scores relate to cognition in SZ. Here we examined the relationship between the 5C-CPT and the CPT-Identical Pairs (CPT-IP), an established and psychometrically robust measure of vigilance from the MATRICS Consensus Cognitive Battery (MCCB) in a sample of SZ patients (*n* = 35). Relationships to global and individual subdomains of cognition were also assessed. 5C-CPT and CPT-IP measures of performance (d-prime) were strongly correlated (*r* = 0.60). In a regression model, the 5C-CPT and CPT-IP collectively accounted for 54% of the total variance in MCCB total scores, and 27.6% of overall cognitive variance was shared between the 5C-CPT and CPT-IP. These results indicate that the reverse translated 5C-CPT and the gold standard CPT-IP index a common attentional construct that also significantly overlaps with variance in general cognitive performance. The use of simple, cross-species validated behavioral indices of attentional/cognitive functioning such as the 5C-CPT could accelerate the development of novel generalized pro-cognitive therapeutics for SZ and related neuropsychiatric disorders.

## Introduction

Attentional dysfunction is a core deficit in schizophrenia (SZ) that negatively impacts functional outcomes^1^. Current pharmacological treatments, however, have limited efficacy for treating cognitive deficits. The link between cognition and functional outcomes, combined with lack-of-effective treatments, has galvanized research to identify pro-cognitive therapeutics for SZ patients^2–6^. While pre-clinical research has yielded insights that may inform the future development of pro-cognitive treatments, profound translational gaps across pre-clinical and clinical studies exist. These gaps remain in large part due to the limited use of cognitive paradigms with cross-species translational validity and relevance^7–9^.

Over the past 15 years the National Institutes of Mental Health (NIMH) has sponsored two projects intended to improve the measurement of cognitive deficits in SZ and identify promising tasks with translational validity. The first, the Measurement and Treatment Research to Improve Cognition in Schizophrenia (MATRICS) initiative, was an effort to standardize cognitive measurement for approval of any pro-cognitive compounds. The product of this initiative, the MATRICS Consensus Cognitive Battery (MCCB), was designed for use in clinical trials and now represents the “gold standard” assessment of cognitive functioning in SZ. Given that the purpose of this battery was to inform clinical trials, limited effort was made to develop this battery in conjunction with cross-species available non-human tests^10^. The Cognitive Neuroscience Treatment Research to Improve Cognition in Schizophrenia (CNTRICS) initiative was established to identify cross-species relevant behavioral paradigms of cognitive constructs such as attention^11^.

The 5-choice continuous performance test (5C-CPT) was developed for use in mice^10^, and was highlighted by the CNTRICS initiative as a promising cross-species paradigm for assessing the control of attention^11^. The 5C-CPT has shown good cross-species validity, including (a) 36 h sleep deprivation-induced deficits^12^; (b) amphetamine-induced improvement^13^; and (c) vigilance decrement observations across time^6,10^. Importantly, patients with SZ exhibit deficient 5C-CPT performance^6,14^, consistent with other CPTs^15,16^. To-date however, no studies have validated the 5C-CPT against a consensus gold standard attentional measurement nor evaluated how 5C-CPT performance is related to cognition in SZ.

Continuous performance tasks (CPTs) are the most common paradigms used for quantifying attentional functioning in neuropsychiatric patients including SZ^15^. In their simplest form, CPTs involve subjects’ being presented with a string of rapidly occurring stimuli and asked to identify targets from among background or non-target stimuli. Common variants of the CPT include CPT-Identical Pairs version (CPT-IP- chosen as part of the MCCB), degraded stimulus CPT (DS-CPT), the AX-CPT (AX-CPT), and the Connors’ CPT (C-CPT). Whereas, all CPTs are designed to quantify sustained attention/vigilance, each CPT emphasizes a unique balance between target detection and response inhibition, which may have important implications for cognition. Important methodological differences also exist across tasks and place demands on other cognitive domains^17^. For example, the CPT-IP has been referred to as a “memory load” CPT given that responses are required when two sequentially presented numbers are identical, likely indexing both attention and working memory. The DS-CPT has been referred to as a “perceptual load CPT” since target stimuli with varying degrees of degradation are presented to subjects and therefore is sensitive to both perceptual and attentional deficits^18,19^. The AX-CPT requires responding when the letter X follows an A in sequence (ignoring B and Y stimuli presentations), likely requiring modest working memory functioning in addition to attentional control^20^. In contrast, the Connors’ CPT simply requires responses when target stimuli (letters other than X) are presented, and response inhibition when non-target stimuli (letter X) are presented^21^. Despite these task differences, CPT paradigms have demonstrated clinical sensitivity in quantifying attentional deficits in first episode and chronic SZ patients, unmedicated SZ patients, and unaffected first-degree relatives of SZ patients^19,22–25^.

Psychometric theories of cognition suggest that cognitive abilities are hierarchically structured such that variance (and covariance) in specific abilities can be explained by a smaller number of general abilities^26^. Attention is thought to represent a core domain of cognitive functioning, which might explain large correlations between measures of attentional performance and overall cognition as measured by the MCCB^27^. Furthermore, attention-dependent cognitive measures have been used as exemplars to demonstrate applicability as important targets of medications used to treat SZ^2^.

Although, CPTs are established measures of attentional functioning, they are often multidimensional (as described above), potentially confounding attentional dysfunction with deficits in perceptual or other cognitive domains, e.g., working memory. Moreover, lack-of-cross-species CPTs further limits opportunities for novel treatment development. Quantifying attentional abilities underlying normal and impaired cognitive performance in SZ patients on the 5C-CPT could accelerate the development of pro-cognitive therapeutics. The availability of tasks for measuring similar attentional abilities across species would enable researchers to develop agents for targeting attentional systems in animals and begin spanning the translational gap to human findings.

This study aimed to characterize SZ patients’ attentional task performance on the reverse-translated 5C-CPT in comparison to a “gold standard” measure, the CPT-IP, and to assess its relationship with a global measure of cognition (MCCB). We hypothesized that measures of 5C-CPT and CPT-IP task performance would be significantly correlated and that attentional functioning (as measured by both CPTs) would predict cognitive test performance (MCCB total score). To determine the effects of symptom severity on attentional and cognitive functioning, we assessed the extent to which 5C-CPT, CPT-IP, and MCCB scores were related to positive and negative symptom ratings.

## Methods

### Participants

Thirty-five SZ patients between the ages of 18–61 years old were recruited from a transitional care facility that primarily serves people with diagnoses of SZ or schizoaffective disorder (Table 1). Exclusion criteria for the study included: history of neurological disease, history of major head injury (loss-of-consciousness >15 min), substance dependence within the last 6 months, severe systemic medical illness (e.g., Hepatitis C, HIV, insulin-dependent diabetes), IQ below 70 as estimated by the reading subtest of the Wide Range Achievement Test (WRAT), and difficulty with hearing, vision or English language comprehension that may interfere with the patient understanding consent, screening questions, and task directions. The Institutional Review Board of University of California, San Diego, approved all experimental procedures (IRB#130874). All participants underwent an informed consent procedure, structured clinical diagnostic assessments including a modified Structured Clinical Interview for DSM-IV Axis I disorders (SCID-I), and the Scales for the Assessment of Positive and Negative Symptoms (SAPS and SANS)^28,29^, and cognitive assessment using the MCCB (the Mayer-Salovey-Caruso Emotional Intelligence Test was not administered due to time limitations). The CPT-IP score was omitted when calculating the MCCB total score to avoid biasing predictive relationships. The MCCB neurocognitive composite score was calculated using the mean of the domain *T*-scores, as is consistent with prior publications^30^. The 5C-CPT was completed following the diagnostic and cognitive assessments.

**Table 1:** Participant demographics

Demographics (±s.d., min–max) (*n* = 35)	
Mean age (yrs)	36.1 (±12.7, 19–61)
Education	12.1 (±2.1, 8–18)
Sex (% male)	51.0%
Smoking	0%^a^
Right handedness	63.9%
Age of onset (yrs)	19.3 (±4.5, 8–30)
Illness duration (yrs)	16.7 (±12.9, 1–47)
SAPS total score	5.14 (±4.7, 0–16)
SANS total score	6.43 (±4.2, 0–16)

^a^All participants were housed within a non-smoking transitional care facility, and were free from nicotine for at least 2 months prior to testing. Although, meta-analytic research demonstrated substantial comorbidity between SZ and nicotine use^46^ more recent research indicated smoking may decrease MCCB performance^47^

### 5-choice continuous performance task (5C-CPT)

**Fig. 1:**
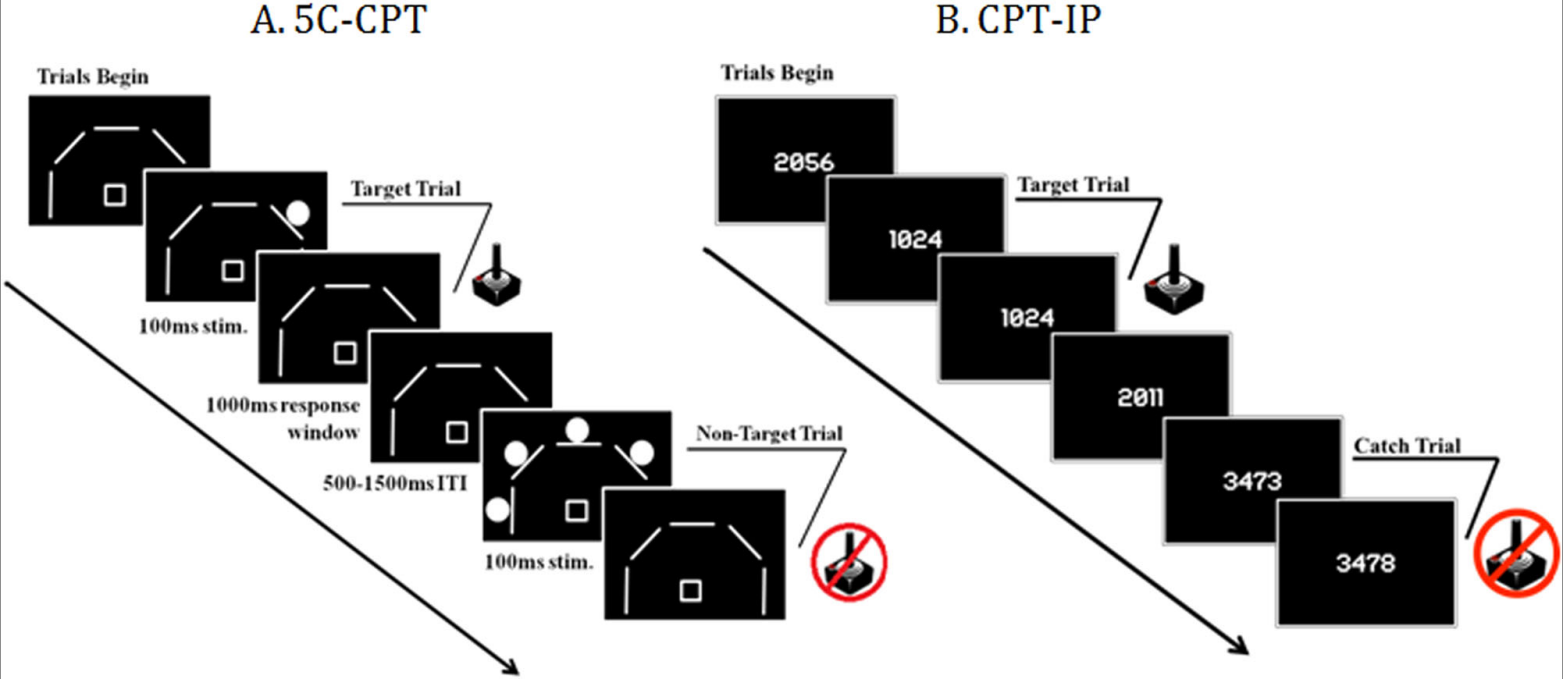
Continuous performance task schematic for the 5C-CPT and CPT-IP. **a** Trial layout for the 5-choice continuous performance task (5C-CPT). **b** Trial layout for the Continuous performance Test-Identical Pairs version (4-digit variant shown). 5C-CPT target trials require responding in the direction of a location in which a single-white circle appears via joystick. Non-target trials require response inhibition when all five white circles appear simultaneously. The CPT-IP requires responding on trails when the same number is presented consecutively (target trials); and response inhibition on all other trials. CPT-IP catch trials require response inhibition when two similar but not identical numbers of presented on consecutive presentations

The 5C-CPT requires responses to targets and inhibition of responses to non-target trials. Participants were instructed to move the joystick in the direction a circle (target stimuli) appears, but inhibit from responding if five circles appeared simultaneously (non-target stimuli) (Fig. 1a)^14^. Stimuli were presented for 100 ms in a random order to reduce temporal predictability with a 1 s response window available and a variable inter-trial interval (ITI; 0.5, 1.0, or 1.5 s). All participants performed a practice block prior to initiating the session and indicated they understood the task. The full task consisted of 270 trials, 225 target and 45 non-target stimuli, presented pseudorandomly so that no >3 presentations of a specific stimulus appeared consecutively. Responses were recorded and include hits and misses to target trials, and false alarms (FAs) and correct rejections (CRs) to non-target trials. Composite metrics of task performance were used in the analysis of performance, including hit rate (HR), false alarm rate (FAR), task accuracy, d′, and responsivity index (RI) as indicated in our previous work^6^.

### Continuous performance task-identical pairs version (CPT-IP)

Developed by Cornblatt et al.^31^, the CPT-IP version is a computerized measure of sustained, focused attention. The task involves monitoring a series of numbers (2, 3, or 4 digits in length) and responding when two identical stimuli occur consecutively (Fig. 1b).

### Statistical analyses

All statistical analyses were conducting using SPSS (IBM Corp., Armonk, NY, USA). Paired sample *t*-tests were used to compare performance metrics between 5C-CPT and CPT-IP. Pearson correlations were used to test relationships between CPT d′ measures, MCCB total score, and symptom ratings (SANS, SAPS). Multivariate linear regression models using 5C-CPT and CPT-IP d′ measures as predictors, were used to determine the unique contribution of each behavioral measure to cognition (MCCB total score). Estimates of variance explained (*R*^2^), standardized regression slopes (β), and Pearson correlations between predictors are reported^32^. Statistical significance for correlations was adjusted for multiple comparisons using the Bonferroni method^33,34^.

## Results

As shown in Table 2, SZ patients exhibited poorer performance on the CPT-IP, demonstrated by significantly lower hit rate (HR), higher false alarm rate (FAR), and lower d′ compared to 5C-CPT performance. There was however, a large positive correlation between 5C-CPT and CPT-IP d′ measures (*r* = 0.60, *p* < 0.001). Additional correlations between each CPT d′, MCCB total score and MCCB subscales are reported in Table 3. The 5C-CPT d′ was significantly and positively correlated with MCCB composite scores, as well as with working memory (WM) and reasoning and problem solving (RPS) subdomain scores, with a trend-level positive relationship with the verbal learning subdomain. The CPT-IP d′ was significantly correlated with MCCB composite score and all cognitive subdomain scores except verbal learning (Table 3). There were weak but non-significant correlations between SANS and SAPS scores and 5C-CPT, CPT-IP, or MCCB total scores (all *r*’s < 0.31, ps > 0.08) (Table 3), consistent with previous reports^19^.

**Table 2:** Behavioral task descriptive statistics

Task/measure	Mean (SEM)	
	**5C-CPT**	**CPT-IP**
d′	3.85 (0.23)	1.85 (0.13)^a^
Hit rate (HR)	0.90 (0.35)	0.68 (0.04)^a^
False alarm rate (FAR)	0.03 (0.01)	0.15 (0.01)^a^
Responsivity index (RI)	−0.23 (0.07)	−0.24 (0.05)
*MCCB (T-scores)*	*Mean (SEM)*	*Range*
Composite score	34.29 (1.2)	18.8–49.4
Speed of processing	30.8 (1.8)	8–55
Visual learning	31.5 (1.9)	14–59
Verbal learning	34.1 (0.9)	21–46
Working memory	33.3 (2.2)	5–55
Reasoning and problem solving	41.7 (1.4)	28–59

Behavioral task performance and MCCB composite and subscale means, standard errors, and response ranges

^a^Indicates *p* < 0.01

**Table 3:** Attention—cognition correlations

	5C-CPT	CPT-IP	Fisher’s z
5C-CPT		0.60*	*p*-values
MCCB composite	0.54*	0.72*	NS
Speed of processing	0.33	0.56*	0.03*
Working memory	0.54*	0.64*	NS
Verbal learning	0.44+	0.41	NS
Visual learning	0.26	0.52*	0.02*
Reasoning and problem solving	0.51*	0.54*	NS
SANS total score	−0.30	−0.15	NS
SAPS total score	−0.28	−0.21	NS

* indicates *p* *<* *.*004

Pearson correlations between 5C-CPT and CPT-IP d′s, MCCB composite (without the CPT-IP included), subscale *T*-scores, and SANS and SAPS total scores. Statistical significance (*) was determined based on a Bonnferoni correction, which required *p* < 0.004. Plus symbol (+) indicates corrected trend-level significance *p* < 0.01. Right column depicts *p*-values for Fisher-z correlation comparisons between left and middle columns using single-sided testing

### Regression analyses

**Fig. 2:**
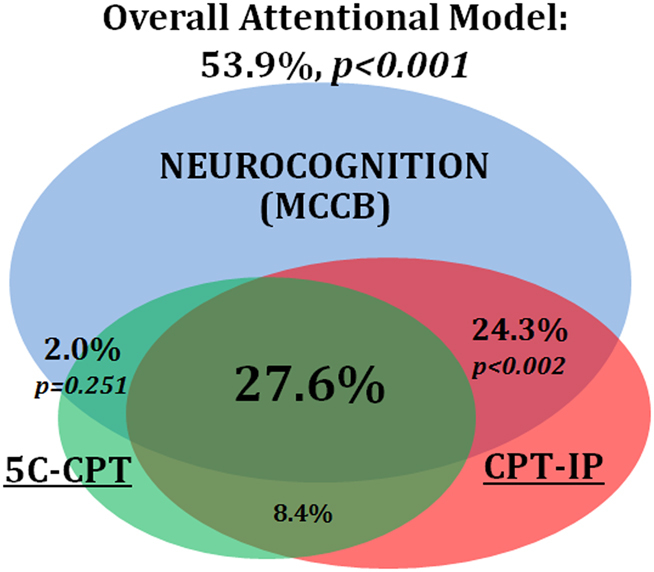
Behavioral measure variance components predicting cognition. Total model variance in cognition (MCCB Total score) accounted for was 53.9%. Outer circles depict the unique variance proportions for each predictor. The CPT-IP accounted for 24.3% of the variance, while the 5C-CPT uniquely accounted for only 2.0%. The variance shared between the 5C-CPT and the CPT-IP accounted for 27.6% of the variance in cognition

To assess the shared and unique contributions of each CPT to cognition, MCCB composite scores were regressed onto 5C-CPT and CPT-IP d′ scores. A model including both CPTs accounted for 53.9% of the variance in MCCB composite score (*F*_(2,32)_ = 18.7, *p* < 0.001). Comparisons of model parameter estimates (standardized regression slopes) indicated that 27.6% of the variance in global MCCB performance was shared attentional variance between the 5C-CPT and CPT-IP. The 5C-CPT further accounted for a unique but non-significant 2.0% of the variance in cognition (*b* = 0.956, *β* = 0.175, *p* = 0.251), while the CPT-IP uniquely accounted for an additional 24.3% of the variance (*b* = 5.62, *β* = 0.616, *p* < 0.001) in MCCB composite scores. A Venn diagram visualizing the relationships among the three variables is shown in Fig. 2.

## Discussion

The present study validates the 5C-CPT as an attentional measure relevant to cognition in SZ through its strong association with the gold standard CPT-IP as indicated by their combined relationship with cognitive test performance, independent of symptomatology. The strong relationship observed between the 5C-CPT and the CPT-IP (*r* = 0.60), and whose combined variance accounted for more than half of the variance MCCB total scores (53.9%), underscores the importance of attentional functioning to cognition. Regression analyses indicated that, of the 36% of variance shared between the 5C-CPT and the CPT-IP, 27.6% is likely attention-specific variance that contributes to global cognition (Fig. 2). The residual, non-attentional variance shared between the 5C-CPT and CPT-IP (8.4%) may partially represent behavioral effort required to complete these tasks^35^, or method variance shared between CPTs not associated with cognition. The considerable variance shared between 5C-CPT, CPT-IP, and MCCB scores likely reflects an attentional construct measured to differing degrees by all three tasks.

The 5C-CPT primarily measures attentional functioning similarly to the CPT-IP but with important differences. This is evidenced by the differing relationships between each CPT and the MCCB composite score. After the 27% variance shared between the CPTs and MCCB was accounted for, the residual 2.0% of shared variance between 5C-CPT and MCCB was no longer significant (*p* = 0.25) (Fig. 2). This finding represents a strength of the 5C-CPT as it suggests that the measure may provide a relatively more specific index of attention compared to the gold-standard CPT-IP. The CPT-IP, in contrast, accounted for an additional 24.3% (*p* < 0.001) of the variance in MCCB performance after accounting for shared variance with 5C-CPT, indicating that CPT-IP performance may reflect not only attentional ability but also broader cognitive functioning. This finding is further supported by significant correlations between CPT-IP performance and MCCB subdomains of speed of processing and visual learning (Table 3). The relationships among these domains with CPT-IP performance likely reflect the additional CPT-IP task demands of processing rapidly presented numerical strings and evaluating each as a potential target for comparison to recently presented stimuli. The cognitive resources required for rapid evaluation and matching of numerical strings likely involves resources beyond attention and are thus reflected in the non-attentional relationship between CPT-IP and neurocognition. In contrast, the 5C-CPT does not place such demands on speed of processing, likely reducing its link to this domain. As the relationships between symptom ratings and attentional or cognitive test performance did not survive correction, it is thought current symptom levels minimally affected the relationship between CPT and MCCB performance.

The direct comparison of 5C-CPT and CPT-IP performance enables cross-task validation the 5C-CPT compared to the gold standard CPT-IP. Patient performance across all 5C-CPT performance metrics (HR, FAR, d′) were significantly better than those of the CPT-IP, suggesting that 5C-CPT may generally be less difficult than CPT-IP. The CPT-IP explicitly manipulates task difficulty across blocks using two-, three-, or four-digit strings, compared to the single difficulty level of the 5C-CPT. Supplementary analyses revealed a linear decrease in d′ with increased difficulty on the CPT-IP, consistent with prior reports^36^. The CPT-IP two-digit condition, compared to the three- and four-digit conditions, was most strongly correlated with 5C-CPT, suggesting that this condition most closely approximates the difficulty of the 5C-CPT (Supplemental figure S1). To test the specificity of the relationship between the CPT-IP-2-digit version and the 5C-CPT, all statistics were rerun using only the CPT-IP-2-digit version. the overall model and relationships between cognition and each CPT did not significantly change (Supplemental Material). A recent study comparing four versions of the CPT-IP found no increased sensitivity between versions in SZ patients^36^. Therefore, we aggregated scores across CPT-IP difficulty levels to provide a task-level metric of attentional functioning to compare to the single difficulty of the 5C-CPT. Regardless of this difference in score calculation, the CPT-IP and 5C-CPT shared significant variance and were both significantly related to MCCB performance, underscoring their shared measurement of attention and its relationship to cognition. The current results demonstrate the reverse translated 5C-CPT measures attentional functioning similarly to the gold standard CPT-IP, and can significantly predict cognition in SZ patients in a real world treatment setting.

Attentional functioning (in both CPTs) was also significantly correlated with other cognitive subdomains, and outlines attentional functioning as a necessary but insufficient construct underlying global cognition. Recent research has indicated the importance of attentional functioning for other cognitive processes (i.e., processing speed, planning, reasoning, and problem solving), as well as its direct and indirect relationships with functional outcomes in SZ^11,37,38^. The current data suggest good convergent validity among CPTs for attention measures, but also highlights the challenge of measuring a cognitive domain in isolation. The pattern of correlations between the 5C-CPT, CPT-IP, and MCCB subdomains indicates increased specificity in attentional measurement between the 5C-CPT and cognition compared to the gold standard CPT-IP. In contrast, correlations between CPTs and other cognitive domains ranged from small to moderate (Table 3). Recent neuropsychological findings in adult SZ patients indicate potential gender effects for attention and reasoning/problem solving specifically^39^. While the current study lacked the power to investigate gender specific effects on cognition, they may help explain the moderate correlations between the 5C-CPT and the reasoning and problem-solving subdomain. The relationship between attention and other cognitive subdomains in the current study was present in both CPTs, most notably with WM. Recent research has indicated a detrimental interaction between WM and attention where SZ patients demonstrate disproportional attentional impairment under increasing WM load compared to healthy controls^40^. Additionally, deficits in attention and WM may also be the product of a narrowing of attentional focus compared to controls, accounting for reported deficits in both domains^41^. As SZ patients display deficits across cognitive domains including WM and attentional functioning, the challenge persists on how to accurately quantify attentional functioning in the absence of other cognitive measures. Unfortunately, common CPT variants (CPT-IP, DS-CPT, AX-CPT, Connor’s CPT) all suffer from various levels of interpretive ambiguity by including additional cognitive task demands in the measurement of attentional functioning^2,17^. As attention is thought to be a necessary component of higher-order cognitive functioning, inter-correlations amongst related cognitive domains are to be expected. Thus, future research should utilize reverse-translated tasks targeting specific cognitive domains to better disentangle specific cognitive deficits in psychiatric patients. Given the availability of the mouse^42^ and rat^43,44^ 5C-CPT versions, treatments that improve task performance in animals could potentially be used to improve global cognitive functioning in humans.

A few study limitations deserve discussion, most notably the lack of a non-psychiatric comparison group. Future studies will investigate both SZ and healthy control samples to ascertain differential behavioral relationships between CPT task performance and cognitive functioning across groups. Additionally, as in the vast majority of SZ studies, all participants were medicated at the time of testing, with most treated on a combination of typical and atypical antipsychotic mediation along with other psychotropics. Although, the heterogeneity of medication regimens in our cross-sectional sample precluded the examination of medication effects, our behavioral measures were still sensitive to cognition with this medication-heterogeneous sample. Nonetheless, we cannot rule out an impact of antipsychotic medications on our findings, and future randomized controlled trials should prospectively examine medication effects on subjects. Finally, it is possible that the overlapping variance between 5C-CPT and CPT-IP represents a latent construct other than attention that is relevant to cognitive test performance in SZ. For example, these tasks may also provide a measurement of cognitive control that has been proposed to be consistently disturbed across psychiatric conditions^45^. Beyond cognitive control, however, given the specific task demands of the 5C-CPT and low correlations with non-attentional MCCB subdomains, it is unlikely that other cognitive abilities account for such high-overlapping variance.

In conclusion, we demonstrate a novel reverse translated behavioral measure of attention is robustly related to an established measure of attention and cognitive test performance in a group of transitionally housed SZ inpatients. By validating novel reverse-translated laboratory measures like the 5C-CPT, together with existing gold standard measures of attention and cognition, we can provide more direct cross-species relationships to aid the development of pro-cognitive therapeutics, and ultimately improve functional outcomes in SZ^5^. This translational approach may provide further utility by identifying individuals likely to benefit from treatment and identifying those who may benefit from additional targeted pharmacological or psychosocial pre-treatments to help boost treatment gains and long-term functional outcomes. Finally, as attentional deficits may be present prior to full disease onset and signal poor functional outcomes, this approach may facilitate early identification of individuals at elevated risk for developing pathologies with primary attentional dysfunction.

## Electronic supplementary material

Suppl Material

Suppl Fig IP2

Supplemental legends
