# Supplementary material for: Reverse translated and gold standard continuous performance tests predict global cognitive performance in schizophrenia
Source: Transl Psychiatry. 2018 Apr 12;8:80. doi: 10.1038/s41398-018-0127-5 (PMC5895589; doi:10.1038/s41398-018-0127-5)
Supplement: Supplementary file 1 — Suppl Material [file 41398_2018_127_MOESM1_ESM.docx]

Supplemental Material


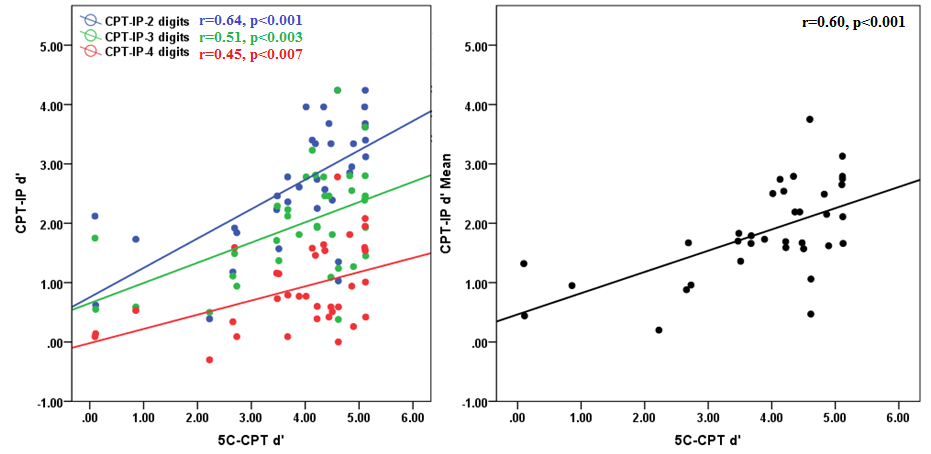


Supplemental figure S1. Scatter plots and correlations between 5C-CPT d' and CPT-IP d' for the (left pane) two-, three-, and four-digit conditions, and the (right pane) collapsed average CPT-IP d'.


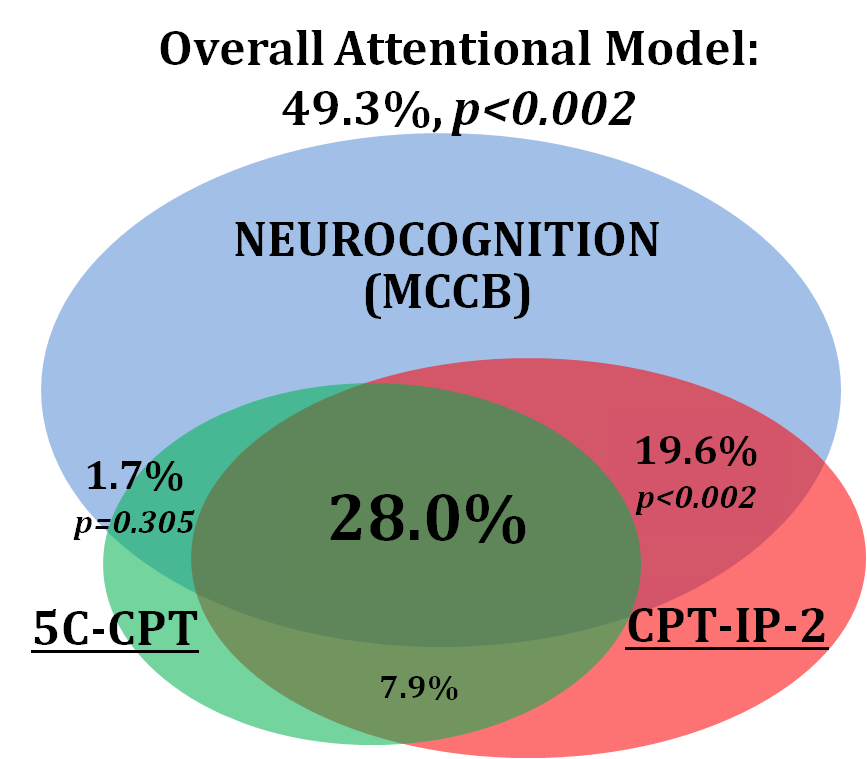


Supplemental Figure 2. Behavioral measure variance components predicting cognition. Total model variance in cognition (MCCB Total score) accounted for was 49.3%. Outer circles depict the unique variance proportions for each predictor. The CPT-IP-2 accounted for 19.6% of the variance, while the 5C-CPT uniquely accounted for only 1.7%. The variance shared between the 5C-CPT and the CPT-IP-2 accounted for 28.0 % of the variance in cognition.

***Supplemental Table 1: Behavioral Task Descriptive Statistics***

| **Task/Measure** | **Mean (SEM)** | |
| --- | --- | --- |
|  | **5C-CPT** | **CPT-IP-2** |
| d' | **3.85 (0.23)** | **2.66 (0.17)*** |
| Hit Rate (HR) | 0.90 (0.35) | 0.79 (0.02)* |
| False Alarm Rate (FAR) | 0.03 (0.01) | 0.07 (0.01) |
| Responsivity Index (RI) | -0.23 (0.07) | -0.71 (0.03)* |
| **MCCB (T-Scores)** | **Mean (SEM)** | **Range** |
| **Composite Score** | **34.29 (1.2)** | **18.8-49.4** |
| Speed of Processing | 30.8 (1.8) | 8-55 |
| Visual Learning | 31.5 (1.9) | 14-59 |
| Verbal Learning | 34.1 (0.9) | 21-46 |
| Working Memory | 33.3 (2.2) | 5-55 |
| Reasoning & Problem Solving | 41.7 (1.4) | 28-59 |

Behavioral task performance and MCCB composite and subscale means, standard errors and response ranges. * indicates *p<0.01*.

***Supplemental Table 2: Attention – Cognition Correlations***

|  | **5C-CPT** | **CPT-IP-2** | **Fisher’s z** |
| --- | --- | --- | --- |
| **5C-CPT** |  | **0.64*** | ***p-values*** |
| **MCCB Composite** | **0.54*** | **0.69*** | *ns* |
| **Speed of Processing** | 0.33 | **0.57*** | **0.01*** |
| **Working Memory** | **0.54*** | **0.64*** | *ns* |
| **Verbal Learning** | 0.44+ | 0.40 | *ns* |
| **Visual Learning** | 0.26 | 0.43 | **0.01*** |
| **Reasoning & Problem Solving** | **0.51*** | **0.50*** | *ns* |
| **SANS Total Score** | -0.30 | -0.14 | *ns* |
| **SAPS Total Score** | -0.28 | -0.23 | *ns* |

Pearson correlations between 5C-CPT and CPT-IP-2 d's, MCCB composite (without the CPT-IP included), subscale T-Scores, and SANS and SAPS total scores. Statistical significance (*) was determined based on a Bonnferoni correction, which required p<0.004. Plus symbol (+) indicates corrected trend-level significance p<0.01. Right column depicts p-values for Fisher-z correlation comparisons between left and middle columns using single-sided testing.
