# Supplementary figures and images for: Reverse translated and gold standard continuous performance tests predict global cognitive performance in schizophrenia
Source: Transl Psychiatry. 2018 Apr 12;8:80. doi: 10.1038/s41398-018-0127-5 (PMC5895589; doi:10.1038/s41398-018-0127-5)

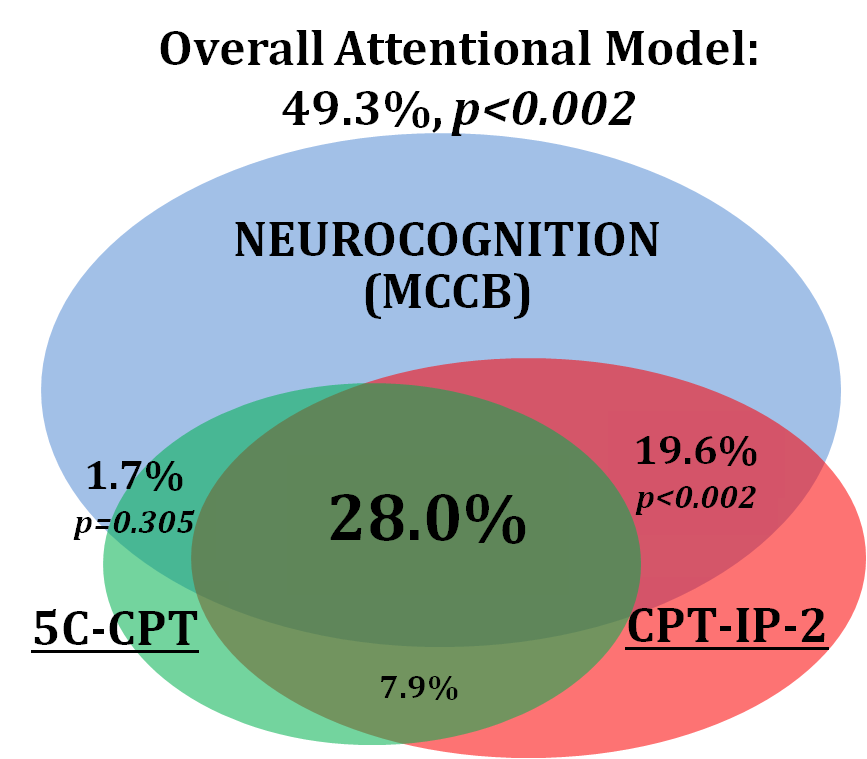

Supplement: Supplementary file 2 — Suppl Fig IP2 [file 41398_2018_127_MOESM2_ESM.tif]
