# Supplementary material for: Reverse translated and gold standard continuous performance tests predict global cognitive performance in schizophrenia
Source: Transl Psychiatry. 2018 Apr 12;8:80. doi: 10.1038/s41398-018-0127-5 (PMC5895589; doi:10.1038/s41398-018-0127-5)
Supplement: Supplementary file 3 — Supplemental legends [file 41398_2018_127_MOESM3_ESM.docx]

Supplemental figure S1. Scatter plots and correlations between 5C-CPT d' and CPT-IP d' for the (left pane) two-, three-, and four-digit conditions, and the (right pane) collapsed average CPT-IP d'.

Supplemental figure S2. Behavioral measure variance components predicting cognition. Total model variance in cognition (MCCB Total score) accounted for was 49.3%. Outer circles depict the unique variance proportions for each predictor. The CPT-IP-2 accounted for 19.6% of the variance, while the 5C-CPT uniquely accounted for only 1.7%. The variance shared between the 5C-CPT and the CPT-IP-2 accounted for 28.0 % of the variance in cognition.

Supplemental Table 1. Behavioral task performance and MCCB composite and subscale means, standard errors and response ranges. * indicates p<0.01.

Supplemental Table 2. Pearson correlations between 5C-CPT and CPT-IP-2 d's, MCCB composite (without the CPT-IP included), subscale T-Scores, and SANS and SAPS total scores. Statistical significance (*) was determined based on a Bonnferoni correction, which required p<0.004. Plus symbol (+) indicates corrected trend-level significance p<0.01. Right column depicts p-values for Fisher-z correlation comparisons between left and middle columns using single-sided testing.
